# CpG methylation regulates allelic expression of *GDF5* by modulating binding of SP1 and SP3 repressor proteins to the osteoarthritis susceptibility SNP rs143383

**DOI:** 10.1007/s00439-014-1447-z

**Published:** 2014-05-27

**Authors:** Louise N. Reynard, Catherine Bui, Catherine M. Syddall, John Loughlin

**Affiliations:** 1 Musculoskeletal Research Group, Institute of Cellular Medicine, 4th Floor Catherine Cookson Building, The Medical School, Framlington Place, Newcastle University, Newcastle upon Tyne, NE2 4HH UK; 2 Present Address: Département de Génétique, Unité INSERM U781, Université Paris Descartes-Sorbonne Paris Cité, Fondation Imagine, Hôpital Necker Enfants Malades, 75015 Paris, France

## Abstract

*GDF5* encodes an extracellular signalling molecule that is essential for normal skeletal development. The rs144383 C to T SNP located in the 5ʹUTR of this gene is functional and has a pleiotropic effect on the musculoskeletal system, being a risk factor for knee-osteoarthritis (OA), congenital hip dysplasia, lumbar disc degeneration and Achilles tendon pathology. rs143383 exerts a joint-wide effect on *GDF5* expression, with expression of the OA-associated T allele being significantly reduced relative to the C allele, termed allelic expression imbalance. We have previously reported that the *GDF5* locus is subject to DNA methylation and that allelic imbalance of rs143383 is mediated by SP1, SP3 and DEAF1 transcriptional repressors. In this study, we have assayed *GDF5* methylation in normal and osteoarthritic cartilage, and investigated the effect of methylation on the allelic imbalance of rs143383. We observed demethylation of the *GDF5* 5ʹUTR in OA knee cartilage relative to both OA (*p* = 0.009) and non-OA (*p* = 0.001) hip cartilage, with the most significant demethylation observed at the highly conserved +37 CpG site located 4 bp upstream of rs143383. Methylation modulates the level and direction of allelic imbalance of rs143383, with methylation of the +37 CpG dinucleotide within the SP1/SP3 binding site having an allele-specific effect on SP1 and SP3 binding. Furthermore, methylation attenuated the repressive effects of SP1, SP3 and DEAF1 on *GDF5* promoter activity. This data suggest that the differential methylation of the +37 CpG site between osteoarthritic hip and knee cartilage may be responsible for the knee-specific effect of rs143383 on OA susceptibility.

**Electronic supplementary material:**

The online version of this article (doi:10.1007/s00439-014-1447-z) contains supplementary material, which is available to authorized users.

## Introduction

Osteoarthritis is a chronic and age-associated disease that severely affects the function of synovial joints, principally the knees, hips, and hands. OA pathogenesis involves the irreversible focal destruction of articular cartilage, with alteration in the homeostatic balance of the cartilage chondrocytes cells, and with accompanying changes in other joint tissues (Houard et al. 2013; Loeser et al. 2012). Pain and disability is a common feature of the disease, with the economic cost of OA estimated to be 1–2 % of gross national product in Western countries (McGuire et al. 2002). The incidence and prevalence of OA will rise over the next few decades as average lifespan and obesity levels continue to increase.

Twin pair and segregation studies have highlighted genetics as a significant risk factor for OA, with heritability estimates as high as 80 % at some joint sites (Loughlin 2005). Genome-wide association scans together with candidate gene studies have identified several genomic regions that harbour OA susceptibility alleles. However, these studies rarely identify which variants are causal, and, like most common diseases, the identification of these alleles and elucidation of the mechanism through which they increase disease susceptibility has proven challenging. The most compelling OA risk allele, and the only one so far that is unambiguously shared between European and Asian individuals, is the rs143383 single nucleotide polymorphism (SNP) on chromosome 20 (Reynard and Loughlin 2013). In Europeans, this association is particularly relevant to knee OA compared to the other joint that has been extensively investigated, the hip (Evangelou et al. 2009).

rs143383 is located within the 5ʹ untranslated region (UTR) of the growth differentiation factor 5 gene *GDF5*, whose protein is a member of the BMP subgroup of the TGF-β superfamily. Mutations of *GDF5* are responsible for several human dominant monogenic disorders including Du Pan syndrome (Szczaluba et al. 2005), Brachydactyly type A2 (Plöger et al. 2008) and multiple synostoses syndrome 2 (Dawson et al. 2006), as well as the mouse brachypodism (bp) phenotype, all of which are characterised by musculoskeletal abnormalities. Mice with targeted overexpression of *GDF5* in cartilage have a reduced chondrocyte proliferative zone and expanded pre-hypertrophic and hypertrophic zones of the growth plate with increased expression of differentiation markers *Ihh* and *Col10a1* (Tsumaki et al. 1999). Overexpression of human *GDF5* in human mesenchymal stem cells enhances chondrocyte differentiation and hypertrophy (Coleman et al. 2013), induces tenogenic differentiation (Tan et al. 2012) and stimulates osteogenesis and bone formation (Cheng et al. 2012). *GDF5* plays an important role in postnatal joint homeostasis and repair (Luyten 1997), and injection of recombinant human *GDF5* into the nucleus pulposus of rabbits after anular puncture of the intervertebral discs is reported to have a reparative effect Chujo et al. 2006). As well as developmental failure of the condyles and intraarticular knee ligament (Harada et al. 2007), GDF5 null mice have delayed cartilage formation and wound callas remodelling after tibial fracture (Coleman et al. 2011). *GDF5* is overexpressed in articular cartilage relative to osteophytic cartilage of OA patients, suggesting that it may play an important role in maintaining the stable articular chondrocyte phenotype (Gelse et al. 2012).

rs143383 is within the *GDF5* promoter and there is an imbalance between the expression of the C and T alleles of this SNP, with an average of 27 % lower expression of the disease-associated T allele than the C allele in synovial joint tissues from OA patients (Southam et al. 2007; Egli et al. 2009). The joint-wide reduction in *GDF5* expression caused by this allelic expression imbalance (AEI) is hypothesised to underlie the OA susceptibility mediated by rs143383 (Southam et al. 2007). In support of this hypothesis, mice with only one copy of *Gdf5* have more severe osteoarthritic changes, including reduced subchondral bone and increased synovial hyperplasia, than their wild-type littermates after mechanical or chemical induction of OA (Daans et al. 2011). The T allele of rs143383 is also associated with several other disease phenotypes, including Achilles tendinopathy (Posthumus et al. 2010), and congenital dislocation of the hip (Rouault et al. 2010), highlighting the pleiotropic action of *GDF5* and its protein in the development, maintenance and repair of the musculoskeletal system.

We have previously reported that DNA methylation regulates *GDF5* expression in human cell lines and that demethylation of the *GDF5* locus is associated with an increase in the allelic imbalance of rs143383 (Reynard et al. 2011). In its C allele form, rs143383 creates a CpG site and is flanked by several non-polymorphic CpG sites, including one that is only four bases upstream of the SNP and at position +37 relative to the *GDF5* transcription start site (TSS). More recently, we demonstrated that the SP1, SP3, SUB1 (also known as P15 and PC4) and DEAF1 *trans*-activating proteins bind to and differentially repress the transcription of the two alleles of rs143383 (Syddall et al. 2013), resulting in the AEI that underlies the OA susceptibility mediated by this SNP. In our current study, we have investigated the interaction between genetics, DNA methylation and transcription at rs143383 using cartilage from OA patients and transformed human cell lines. We examined if *GDF5* was regulated by DNA methylation in cartilage and assessed if this methylation was aberrant in osteoarthritic patients. We observed up-regulation of *GDF5* expression in the cartilage chondrocytes after exposure to a demethylating agent and after the siRNA knock-down of enzymes that methylate DNA, indicating that DNA methylation represses *GDF5* expression in this tissue. Furthermore, bisulphite pyrosequencing revealed that the *GDF5* 5ʹUTR is demethylated in OA knee cartilage and that methylation of this region is associated with a reversal of the imbalance between the two alleles of rs143383. We then investigated the effect DNA methylation has on the binding to and repression of the *GDF5* 5ʹUTR by SP1, SP3 and DEAF1 using electrophoretic mobility shift assays (EMSAs) and luciferase assays. Our data demonstrate that methylation regulates *GDF5* expression in cartilage and modulates the functional effect of the OA SNP rs143383 by altering binding of SP1, SP3 and DEAF1 transcriptional repressors. Our data also show that these effects are particularly striking for knee cartilage relative to hip cartilage, which may partly explain why the rs143383 OA association is more pronounced at the knee.

## Results

### *GDF5* is up-regulated in OA cartilage

**Fig. 1:**
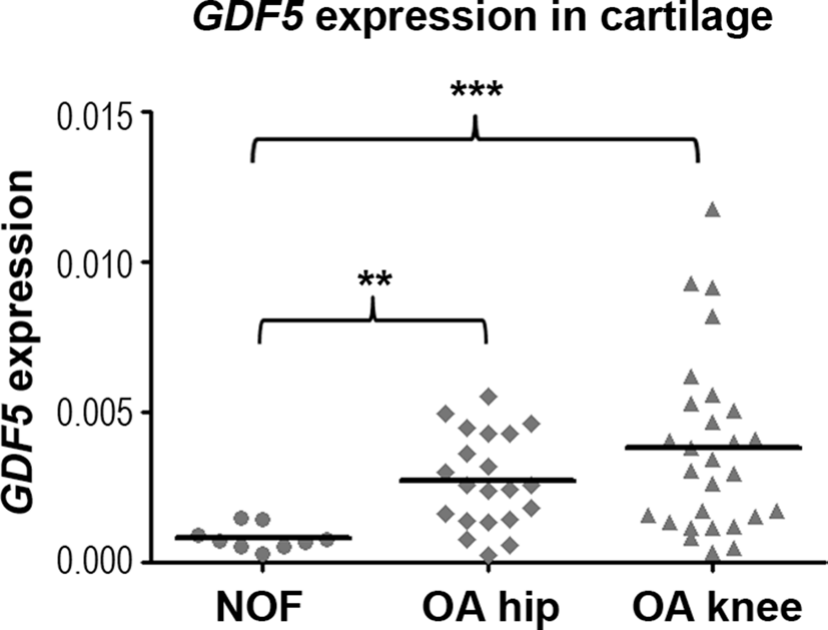
*GDF5* expression in cartilage. *GDF5* expression in cartilage samples from NOF (*n* = 9), OA hip (*n* = 21) and OA knee (*n* = 28) patients was assayed by qRT-PCR and normalised to *18s*. *Bars* represent the mean. **p < 0.01, ***p < 0.00, Mann–Whitney *U* test

We had previously generated gene expression microarray data for hip cartilage from patients who had undergone joint replacement surgery due to either hip OA or a neck of femur (NOF) fracture (Xu et al. 2012). This latter cartilage serves as a non-OA control. Our analysis of *GDF5* revealed that the gene was up-regulated 1.9-fold in cartilage from OA patients (*n* = 12) relative to the control NOF patients (*n* = 13; *p* < 0.0001, Figure S1A). We replicated this result by quantitative real-time PCR (qRT-PCR) analysis of *GDF5* expression in an unrelated sample of nine NOF and 21 OA hip patients, with the gene again showing an up-regulation in OA, this time 3.3-fold (*p* < 0.01, Fig. 1). We also performed *GDF5* qRT-PCR on knee cartilage from 28 OA patients and noted that the gene was also up-regulated in this tissue, 4.6-fold, compared to the non-OA control cartilage (*p* < 0.001, Fig. 1). Overall, *GDF5* showed greater expression in OA than non-OA cartilage, and this effect was particularly striking for the knee. No effect of age, gender or rs143383 genotype on the expression of *GDF5* was observed (Figure S1B-D).

### Inhibition of DNA methylation in articular chondrocytes is accompanied by increased expression of *GDF5*

**Fig. 2:**
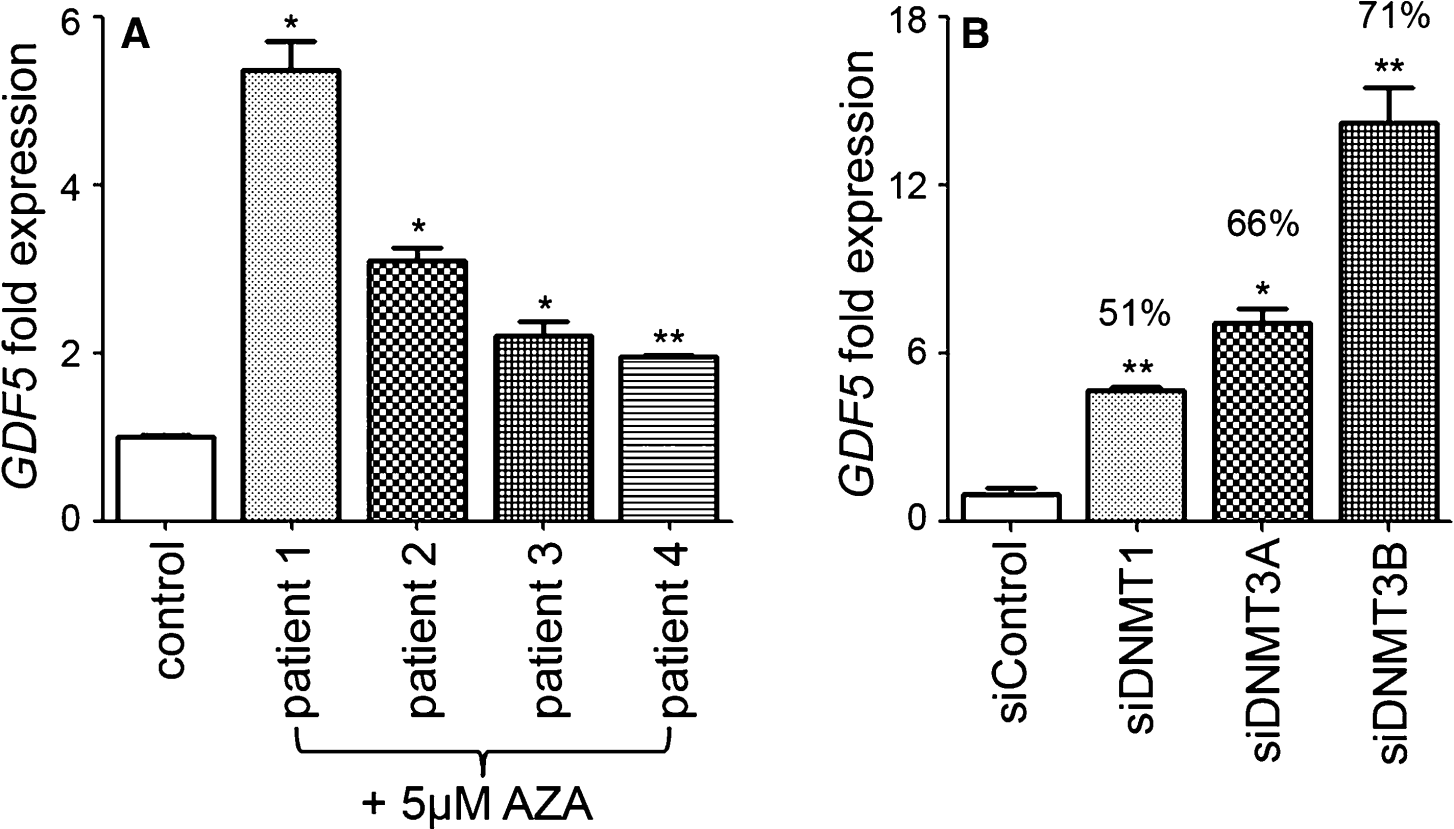
Inhibition of DNA methylation is associated with increased *GDF5* expression in human articular chondrocytes (HACs). **a** Effect of AZA treatment on *GDF5* expression. HACs from four OA patients were cultured in 5 µM AZA dissolved in 50 % acetic acid or 50 % acetic acid alone (control) for three passages and gene expression measured after the third passage. **b** *GDF5* expression in HACs transfected with siRNAs against the DNA methyltransferase enzymes and the non-targeting control siRNA. Four wells of a 96-well plate were transfected per siRNA for three independent experiments. The percentage of siRNA-mediated inhibition of expression relative to the control siRNA is stated for each enzyme. *GDF*5 expression levels were normalised to *18s* and control-treated cells and those transfected with the control siRNA given an arbitrary value of 1. *Error bars* denote standard error of the mean. *p < 0.05, **p < 0.01, Mann–Whitney *U* test

We previously reported that *GDF5* expression is up-regulated in four human transformed cell lines after exposure to the DNA methyltransferase inhibitor 5-Aza-2′-deoxycytidine (AZA), which induced significant demethylation of the *GDF5* locus in these cells (Reynard et al. 2011). To investigate if DNA methylation also regulates *GDF5* expression in cartilage, primary human articular chondrocytes (HACs) from four OA patients were cultured with 5 µM AZA for three passages. *GDF5* expression was significantly increased in HACs from all four patients after AZA treatment (Fig. 2a), with the level of up-regulation varying between patients from 1.95-fold to 5.37-fold. To further examine the effect of DNA methylation on *GDF5* expression in HACs, the DNA methyltransferase enzymes, which catalyse the transfer of methyl groups from S-adenosyl methionine to CpG dinucleotides, were depleted by RNA interference (RNAi; Fig. 2b). There was a significant up-regulation of *GDF5* after depletion of DNMT1 (*p* < 0.01), which methylates hemimethylated DNA after DNA replication, and after depletion of the de novo methyltransferases DNMT3A (*p* = 0.02) and DNMT3B (*p* = 0.03), suggesting that these enzymes are involved in regulating *GDF5* expression in HACs.

### *GDF5* is demethylated in OA cartilage

**Fig. 3:**
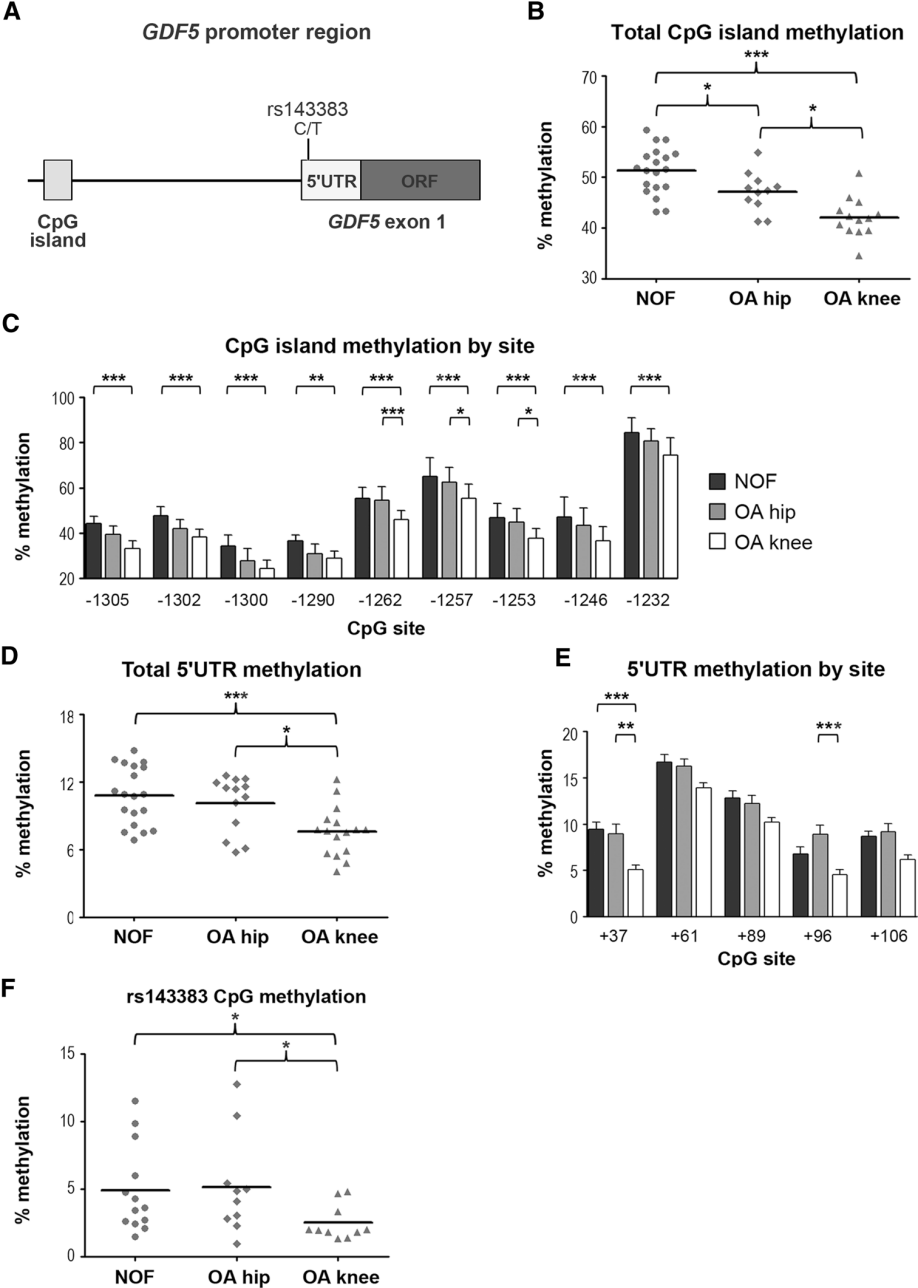
Methylation of the *GDF5* locus in NOF, OA hip and OA knee cartilage. Methylation was assessed by pyrosequencing of bisulphite converted genomic DNA. **a** Schematic diagram of the *GDF5* CpG island and 5ʹUTR. The CpG island spans from −1,345 to −1,210 bp upstream of the *GDF5* TSS and contains nice CpG sites. The 5ʹUTR is located from +1 to +315 bp. **b** Percentage overall methylation of the CpG island in cartilage from NOF (*n* = 19), OA hip (*n* = 11) and OA knee (*n* = 13) patients. **c** Methylation of the CpG island by CpG site. **d** Total methylation of five CpG sites within the 5ʹUTR in cartilage from NOF (*n* = 19), OA hip (*n* = 13) and OA knee (*n* = 16) patients. **e** Methylation of the 5ʹUTR by CpG site. **f** Methylation of the CpG site created by the C allele of rs143383 in cartilage from patients homozygous (CC) or heterozygous (CT) for this allele. NOF = 11 CT and 2 CC; OA hip = 8 CT and 2 CC; OA knee = 8 CT and 2 CC. *Bars* in **b**, **d** and **f** represent the mean and the *error bars* in **c** and **e** denote standard deviation. *p < 0.05, **p < 0.01, ***p < 0.001, one-way ANOVA with Bonferroni correction for multiple testing

The above result demonstrated that DNA methylation modulates *GDF5* expression in cartilage, and our previous study demonstrated that methylation of the CpG island and 5ʹUTR significantly represses the promoter activity of these regions in vitro (Reynard et al. 2011). We therefore investigated if the increased expression of *GDF5* in OA cartilage that we observed in Fig. 1 was due to demethylation of the *GDF5* locus. We analysed the methylation of the CpG island and 5ʹUTR in cartilage obtained from NOF, OA hip and OA knee patients. The CpG island is located between −1,345 and −1,210 bp upstream of the *GDF5* transcription start site (TSS; Fig. 3a), and was significantly demethylated in OA hip (*n* = 11, *p* = 0.02) and OA knee cartilage (*n* = 13, *p* < 0.0001) relative to non-OA NOF cartilage (*n* = 19, Figs. 3b, S2B and S2C). OA knee cartilage had the lowest levels of methylation at all nine CpG sites within the CpG island relative to OA hip and NOF cartilage, and there was significant demethylation at −1,262, −1,257, and −1,253 bp CpG sites in OA hip versus OA knee cartilage (*p* < 0.01, Fig. 3c). The 5ʹUTR region spanning +37 to +106 bp downstream of the TSS was also hypomethylated in OA knee cartilage relative to NOF hip cartilage (*p* < 0.0001) and relative to OA hip cartilage (*p* = 0.025; Figs. 3d, S2D and S2E), with significant demethylation at the +37 CpG site (Fig. 3e). No difference in methylation of the 5ʹUTR was observed in OA hip cartilage relative to non-OA hip cartilage at any of the non-polymorphic CpG sites within this region. In addition, methylation of the CpG site created by the C allele of rs143383 was significantly reduced in knee cartilage relative to hip cartilage from individuals homozygous or heterozygous for the C allele (*p* < 0.05, Fig. 3f). Bearing in mind that the OA association to rs143383 is more pronounced in knee than in hip OA (Evangelou et al. 2009), these two last observations appear highly relevant. There was no effect of gender or rs143383 genotype on mean methylation of the CpG island (Figure S2B, S2C and S2F) or the 5ʹUTR (Figure S2D, S2E and S2G), nor for any individual CpG site within these regions (data not shown). Together, these data demonstrate that *GDF5* is hypomethylated in OA cartilage and this is markedly evident within the 5ʹUTR for OA knee cartilage.

### Methylation of the 5ʹUTR modulates the direction of rs143383 allelic imbalance

**Fig. 4:**
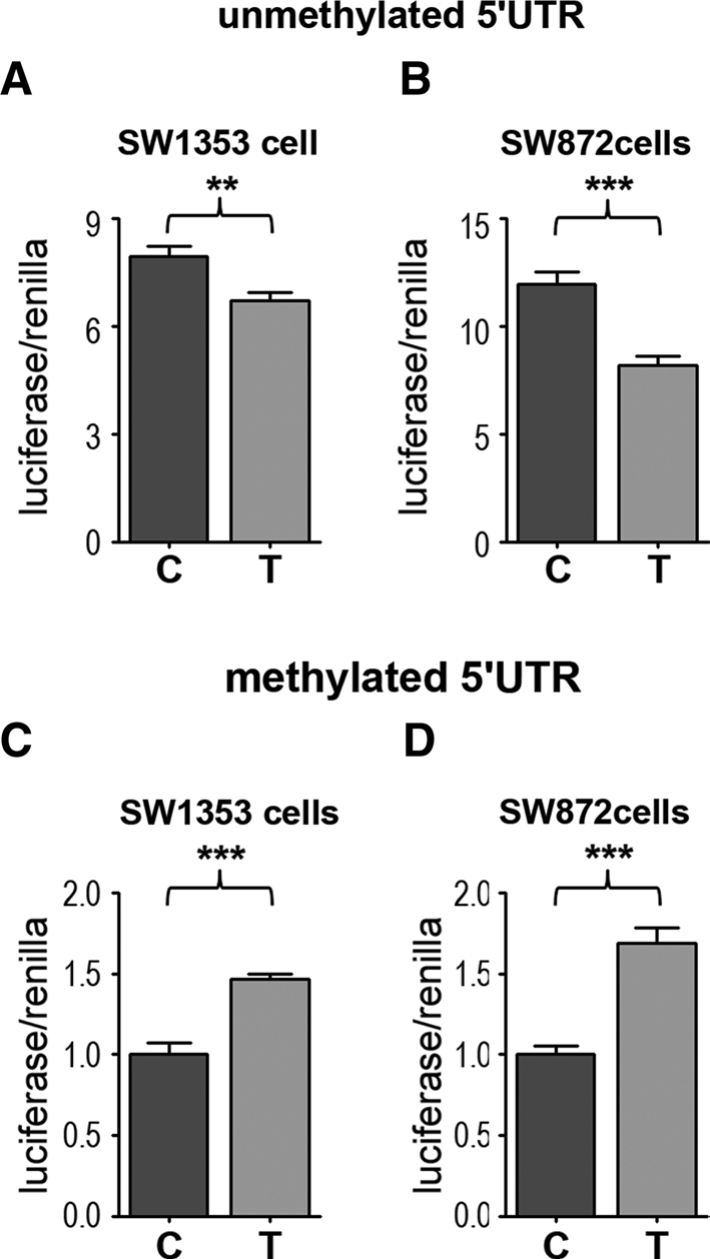
Effect of DNA methylation on the promoter activity of *GDF5* 5ʹUTR. **a** and **b** Luciferase activity of unmethylated pCpGL-5ʹUTR plasmids with either a T allele or C allele at rs143383 after transfection into (**a**) SW1353 chondrosarcoma and (**b**) SW872 liposarcoma cells. **c** and **d** Promoter activity of in vitro methylated pCpGL-5ʹUTR transfected into (**c**) SW1353 cells and (**d**) SW872 cells. Luciferase values were normalised to *Renilla* activity and data shown are the mean ± standard deviation for three independent experiments (*n* = 6) per cell line. **p < 0.01, ***p < 0.001, Mann–Whitney *U* test

We have previously shown that the AEI of rs143383 is increased after AZA induced demethylation of *GDF5* in the heterozygous SW872 human liposarcoma cell line (Reynard et al. 2011). However, AZA globally demethylates DNA and so this may be an indirect effect due to AZA-induced expression changes in *trans*-acting factors that regulate the allelic imbalance rather than demethylation of *GDF5*. To further explore how DNA methylation modulates the genetic effect of rs143383, we analysed the effect of 5ʹUTR methylation on the imbalance between the C and T alleles. The 5ʹUTR was cloned into the CpG-free luciferase vector pCpGL, in vitro methylated and transfected into SW1353 human chondrosarcoma and SW872 cells. When unmethylated, the C allele pCpGL-5ʹUTR plasmid had significantly higher luciferase activity relative to the T allele in SW1353 (*C*/*T* ratio of 1.18, *p* = 0.0026, Fig. 4a) and SW872 (*C*/*T* ratio of 1.46, *p* = 0.0003, Fig. 4b) cell lines, similar to the level of allelic imbalance of rs143383 observed in synovial joint tissues (Egli et al. 2009). DNA methylation had a larger repressive effect on the promoter activity of the C allele pCpGL-5ʹUTR construct than the T allele construct, resulting in the inverted *C*/*T* allelic ratio of 0.7 in SW1353 (*p* < 0.0001, Fig. 4c) and 0.59 in SW872 cells (*p* < 0.0001, Fig. 4d).

### Methylation affects the binding of SP1 and SP3 to rs143383 in an allele-specific manner

**Fig. 5:**
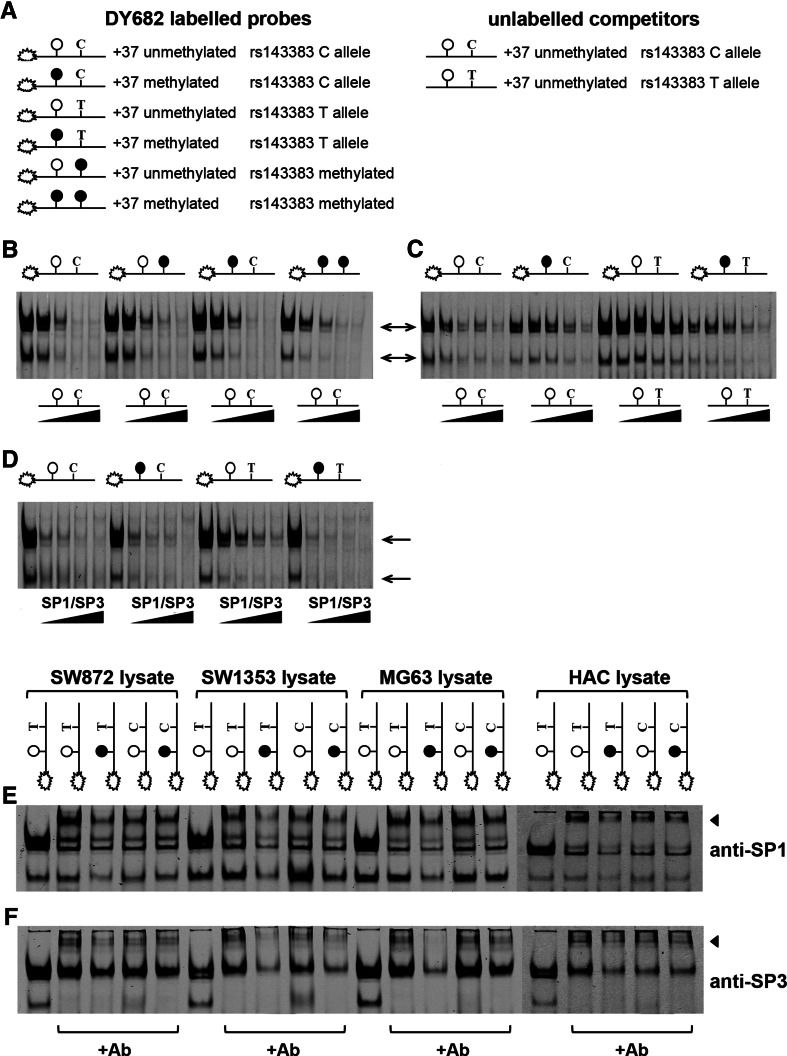
Methylation of the +37 CpG site affects binding of SP1 and SP3 proteins to rs143383. **a** Schematic diagram of the double-stranded DY682-labelled rs143383 probes and unlabelled rs143383 competitors used for EMSA. Open circles represent unmethylated CpG dinucleotides and solid circles represent methylated CpG sites. **b** Effect of methylation on protein binding to the C allele of rs143383. Five, 10-, 25- and 50-fold molar excess of unlabelled C allele competitor was added to the binding reaction as indicated. **c** Effect of +37 CpG methylation on formation of rs143383 T allele and C allele protein-DNA complexes. **d** Competition EMSAs with double-stranded oligonucleotides containing the consensus sequence for SP1/SP3. For B-D, EMSAs were performed with nuclear extracts from the SW872 cell line, which is heterozygous for rs143383, whilst the arrows indicate the rs143383-protein complexes that are formed. **e** and **f** SP1 and SP3 supershift EMSAs. Addition of (**e**) an anti-SP1 and (**f**) an anti-SP3 antibody resulted in supershifting of the rs143383-protein complexes (indicated by *arrowheads*) relative to a control antibody in SW872, SW1353 chondrosarcoma, MG63 osteosarcoma and HACs nuclear protein lysates

To elucidate how DNA methylation modulates the level and direction of allelic imbalance of rs143383, we examined the effect that methylation of the +37 CpG site has on transcription factor binding to rs143383. This CpG site is located 4 bp upstream of rs143383 within the SP1/SP3 consensus site and is demethylated in SW872 cells after AZA treatment concomitant with increased allelic imbalance of rs143383 (Reynard et al. 2011). EMSAs were performed with double-stranded probes encompassing the +37 CpG site and rs143383, and unlabeled unmethylated competitor C and T allele oligonucleotides (Fig. 5a). A lower DNA–protein complex and two upper-protein complexes were observed with both unmethylated C and T allele probes in nuclear lysates from the SW872 cells (arrows in Fig. 5b–d) and we have previously shown that these complexes contain the SP1 and SP3 transcription factors (Syddall et al. 2013). In agreement with our previous report, we found that the protein complexes bound more strongly to the unmethylated T allele probe of rs143383 than the C allele probe (Figure S2A). This was also observed when nuclear extracts from HACs as well as SW1353 chondrosarcoma and MG63 osteosarcoma cell lines were used (data not shown). Methylation of the C allele of rs143383 and +37 CpG site alone or in combination had little or no effect on protein complex binding to the C allele probe (Fig. 5b), with the same concentration of unlabelled unmethylated C allele competitor required to outcompete binding of all four probes. However, methylation of the +37 CpG site did affect the avidity of protein binding to the T allele of rs143383 (Fig. 5c), with a higher concentration of unmethylated T allele competitor required to inhibit the formation of the unmethylated DNA–protein complexes relative to the methylated DNA–protein complexes. This effect of +37 methylation on binding to the T allele probe was also observed when an SP1/SP3 consensus competitor was used, with binding to the methylated T probe outcompeted at a lower competitor concentration relative to the unmethylated T probe (Fig. 5d). Furthermore, the supershifted protein complexes formed upon addition of an anti-SP1 antibody (Fig. 5e) or anti-SP3 antibody (Fig. 5f) were fainter when the methylated T allele probe was incubated with SW872, SW1353, MG63 or HAC nuclear extracts compared to the supershifted complexes formed with the unmethylated T allele probe. Together these data indicate that SP1 and SP3 bind more avidly to the T allele of rs143383 in vitro when the +37 CpG site is unmethylated than when this site is methylated.

### SP1, SP3, SUB1 and DEAF1 repress *GDF5* promoter activity in an allele- and methylation-dependent manner

We have previously shown that SP1, SP3, and DEAF1 repress *GDF5* expression, with the T allele of rs143383 being repressed more than the C allele, leading to the AEI observed at rs143383 (Syddall et al. 2013). Given that methylation of the 5ʹUTR alters the direction of this allelic imbalance and that methylation of the +37 CpG site within the SP1/SP3 consensus sequence abrogates binding of these proteins to the T allele of rs143383 in vitro, as shown above, the role of methylation in allele-specific repression of rs143383 by SP1, SP3 and DEAF1 was further examined. SW1353 cells were co-transfected with a methylated or unmethylated 5ʹUTR pGL3 enhancer luciferase plasmid, an EGFP vector containing the open reading frames (ORF) of SP1, SP3 or DEAF1 and the pTK-RL *Renilla* plasmid. The luciferase readings were normalised against *Renilla* and plotted as a percentage of the values obtained from the co-transfection of the relevant 5ʹUTR plasmid with the empty EGFP vector.

**Fig. 6:**
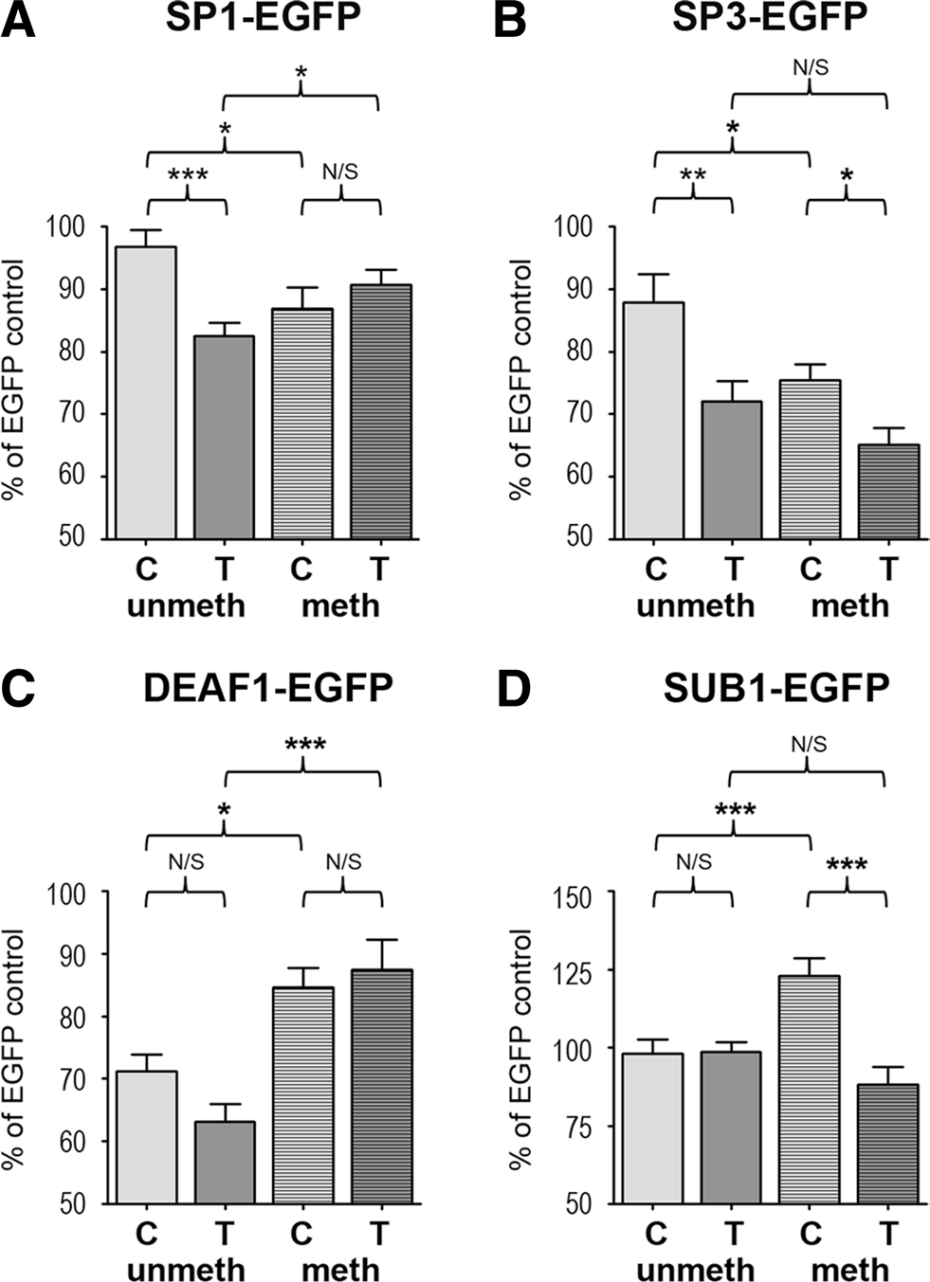
Methylation status influences the effect SP1, SP3, SUB1 and DEAF1 have on promoter activity. Unmethylated and in vitro methylated C luciferase vectors containing the *GDF5* C or T allele 5ʹUTR were cotransfected into SW1353 cells with (**a**) SP1-EGFP, (**b**) SP3-EGFP, (**c**) SUB1-EGFP or (**d**) DEAF1-EGFP plasmids and the control pRL-TK *Renilla* plasmid. Luciferase values were normalised to *Renilla* and are plotted as a percentage relative to the control co-transfection of the empty EGFP plasmid with the relevant unmethylated or methylated C or T allele 5ʹUTR vector. Data shown are the mean ± standard error for four independent experiments (*n* = 5) per cell line. A 2-way ANOVA was performed to test for methylation, differences between the alleles and their interaction. Pairwise comparisons were performed using Fisher’s LSD test to compare the four groups, *p < 0.05, **p < 0.01, ***p < 0.001

There was a statistically significant interaction between plasmid methylation and genotype on the effect of SP1 overexpression on *GDF5* 5ʹUTR promoter activity (*F*_1,55_ = 11.55, *p* = 0.001). As expected based on the EMSA results, SP1 overexpression repressed the unmethylated T allele more strongly than the unmethylated C allele (*p* < 0.0001; Fig. 6a). However, when the 5ʹUTR was methylated, SP1 repressed both alleles to approximately the same extent and had no effect on the allelic ratio. Methylation had a differential effect on the two alleles, with reduced repression of the methylated T allele relative to the unmethylated T allele by SP1 overexpression (91 vs 82 %, *p* = 0.033) and increased repression of the C allele by SP1 when methylated (87 vs 97 %, *p* = 0.011).

There was no significant interaction between methylation and genotype when SP3 was overexpressed, although both methylation (*F*_1,52_, *p* = 0.006) and genotype (*F*_1,52_, *p* < 0.0001) had a significant effect on promoter activity individually. SP3 overexpression significantly decreased the promoter activity of both alleles when the 5ʹUTR was unmethylated, with greater repression of the T allele than the C allele plasmid (*p* = 0.002; Fig. 6b). Surprisingly, the promoter activity of both alleles was more strongly repressed by SP3 overexpression when methylated (*C* = 75 % and *T* = 65 % when methylated, and *C* = 88 % and *T* = 72 % when unmethylated), but this was not significant for the T allele. Although the T allele was more strongly repressed by SP3 irrespective of methylation status, the differential repression of the two alleles was diminished by methylation.

The strongest repression of the unmethylated 5ʹUTR promoter activity was observed when DEAF1 was overexpressed, with the T allele preferentially repressed (Fig. 6c). Methylation significantly reduced the DEAF1-mediated repression of the *GDF5* 5ʹUTR (*F*_1,128_, *p* < 0.0001), and methylation abrogated the differential repression of the C and T alleles.

We previously reported that the transcriptional co-activator SUB1 (P15) binds to the *GDF5* 5ʹUTR and interacts with SP1, SP3 and DEAF1 (Syddall et al. 2013). Although modulating SUB1 protein levels had no effect on *GDF5* expression in that report, we had hypothesised that it acts as a linker between the SP1/SP3/DEAF1 complex and the general transcriptional machinery. We thus examined here what effect SUB1 had on promoter activity of the 5ʹUTR when this region is methylated. This revealed that there was a significant interaction between methylation and genotype on the effect of SUB1 overexpression (*F*_1,118_ = 13.22, *p* < 0.0001). Confirming our previous observations, SUB1 had no effect on the promoter activity of the unmethylated 5ʹUTR plasmids irrespective of genotype (Fig. 6d). However, when the 5ʹUTR was methylated, SUB1 overexpression differentially regulated the two alleles of rs143383, increasing the promoter activity of the C allele by 25 % (*p* < 0.0001) and suppressing the activity of the T allele plasmid.

These results clearly demonstrated that the impact that the four *trans*-acting factors have on gene expression at the allelic level is modulated by the methylation status of the *GDF5* 5ʹUTR.

## Materials and methods

### Tissue collection

Macroscopically normal articular cartilage samples were obtained from patients with NOF fracture with no history of OA and from OA patients undergoing joint replacement of the hip (total hip replacement, THR) or the knee (total knee replacement, TKR). The radiological stage of OA was a Kellgren and Lawrence grade of 2 or more. Cases of inflammatory arthritis, post-traumatic or post-septic arthritis were excluded. No cases suggestive of skeletal dysplasia or developmental dysplasia were included. Tissue was obtained with informed donor consent and Newcastle and North Tyneside Ethics Committee approval (REC reference number 09/H0906/72).

### Human chondrocyte isolation, AZA treatment and RNA interference

Primary human chondrocytes (HACs) were isolated by enzymatic digestion of OA cartilage and cultured as described previously (Bui et al. 2012). Cells were seeded in 25 cm^2^ flasks, and were cultured for one population doubling before being cultured in the presence of 5 µM 5-Aza-2ʹdeoxycytine (AZA; Sigma–Aldrich) dissolved in 50 % acetic acid or vehicle alone (50 %v/v acetic acid) for three population doublings. Fresh AZA was added to the cultures every 2 days during the time course of the experiment. At the end of each population doubling, cells were washed once in PBS and half of the cells harvested for nucleic acid extraction (see below). For siRNA experiments, 10,000 HACs were seeded per well of a 96-well plate overnight and then transfected with SmartPool siRNAs targeted against *DNMT1* (Dharmocon, Thermo Scientific, UK), *DNMT3A* (Qiagen), *DNMT3B* (Dharmocon) and SiControl (Dharmocon) as described by Zhang et al., 2008. Three days post transfection cDNA was synthesised using the Cells-to-cDNA II kit (Invitrogen) and *GDF5* expression was assessed by quantitative real-time RT-PCR as described below.

### Nucleic acid extraction

Following AZA treatment, nucleic acids were extracted from cells grown in monolayer using the Omega EZNA Total DNA/RNA isolation kit (R6731-02; Omega Bio-Tek) following the manufacturer’s instructions. Cartilage samples were collected, snap-frozen in liquid nitrogen and stored at −80 °C until nucleic acid extraction. After frozen tissue was ground under liquid nitrogen using a Retsch mixer mill 200 (Retsch Limited, Leeds, UK), genomic DNA and RNA were extracted as above using a protocol established for human tissue.

### qRT-PCR and genotyping

Total RNA was reverse transcribed using the SuperScript First-Strand Synthesis System (Invitrogen) and *GDF5* expression analysed by quantitative real-time RT-PCR. *GDF5* expression was normalised to the level of *18S* gene expression using the calculation 2^−ΔCt^. Genotyping of rs143383 was performed on cartilage DNA samples using a TaqMan SNP Genotyping Assay (Applied Biosystems). Primers are listed in Table 1.

**Table 1:** List of primers and probes used in this study

Method	Primer sequence
Pyrosequencing	
*CpG island*	FP: 5ʹ-TGTGTTAAGTTGTTTAGGGGTTTTA-3ʹ
	RP: 5ʹ-ACTACAATCTCTACCTCCCAAATTC-biotin-3ʹ
	SP: 5ʹ-AATATTATGTGGGAAATTGT-3ʹ
*5ʹUTR*	FP: 5ʹ-TTGGAGTATATAGGTAGTATTA-3ʹ
	RP: 5ʹ-TCCCCTAAAATCTCTAAC-biotin-3ʹ
	SP: 5ʹ-TTAGTTGTGTAGGAGAAAGG-3ʹ
*GDF5* expression	FP: 5ʹ-CTGTGATTCCAGGAGTGCAG-3ʹ
	RP: 5ʹ-ATCCTCTTCATTGACTCTGCC-3ʹ
	Pr: 5ʹ-CCACGACCATGTCCTC-3ʹ
rs143383 genotyping	FP: 5ʹ-AGTCAGTTGTGCAGGAGAAAGG-3ʹ
	RP: 5ʹ-GCAGCTGAAAATAACTCGTTCTTGAA-3ʹ
	Pr: 5ʹ-AGAAAGCCACCGCC-3ʹ
DY-682 EMSA probes	
rs143383 C allele unmethylated	FP: 5ʹ-GAGAAAGGGGGCGGT**C**GGCTTTCTCC-3ʹ
	RP: 5ʹ-GGAGAAAGCC**G**ACCGCCCCCTTTCTC-3ʹ
rs143383 C allele methylated	FP: 5ʹ-GAGAAAGGGGGC^me^GGT**C**GGCTTTCTCC-3ʹ
	RP: 5ʹ-GGAGAAAGCC**G**ACC^me^GCCCCCTTTCTC-3ʹ
rs143383 T allele unmethylated	FP: 5ʹ-GAGAAAGGGGGCGGT**T**GGCTTTCTCC-3ʹ
	RP: 5ʹ-GGAGAAAGCC**A**ACCGCCCCCTTTCTC-3ʹ
rs143383 T allele methylated	FP: 5ʹ-GAGAAAGGGGGC^me^GGT**T**GGCTTTCTCC-3ʹ
	RP: 5ʹ-GGAGAAAGCC**A**ACC^me^GCCCCCTTTCTC-3ʹ
SP1/SP3 consensus competitor	FP: 5ʹ-AATTGGGGGGGCGGGGGTACGTAGCA-3ʹ
	RP: 5ʹ-TGCTACGTACCCCCGCCCCCCCAATT-3ʹ
Cloning into pGL3-enhancer	FP: 5ʹ-GGGGACTAGTGGATTCAAAACTAGGGGG-3ʹ
	RP: 5ʹ-GGGGAAGCTTCCGCTGAATGACACCAAAG-3ʹ
Cloning into EGFP-N1	
SP1	FP: 5ʹ-GGGGGAATTCATGGATGAAATGACAGCTGTG-3ʹ
	RP: 5ʹ-GGGGCCGCGGGAAGCCATTGCCACTGATATT-3ʹ
SP3	FP: 5ʹ-GGGGGAATTCATGACCGCTCCCGAAAAGCCC-3ʹ
	RP: 5ʹ-GGGGCCGCGGCTCCATTGTCTCATTTCCAGA-3ʹ

The bold and underlined bases in the EMSA probes indicate the position of rs143383. The series of underlined bases in the SP1/SP3 competitors indicate the site of the consensus sequence for SP1/SP3

*FP* forward primer, *RP* reverse primer, *SP* sequencing primer, *Pr* probe, *me* methylated

### DNA methylation analysis by pyrosequencing

Cartilage genomic DNA was bisulphite converted using the EpiTect Bisulfite kit (Qiagen) according to the manufacturer’s instructions. The methylation status of the *GDF5* CpG island and 5ʹUTR was assayed by quantitative bisulphite pyrosequencing of PCR-amplified regions as previously described (Reynard et al. 2011). Two independent PCR replicates were performed per sample and the biotin-labelled products sequenced using the PyroMark Q96 MD system. Pyro Q CpG 1.0.6 software was used to exclude samples that did not pass the bisulphite conversion criteria and to quantify methylation.

### pCpGL in vitro methylation and transient transfection

The *GDF5*-pCpGL plasmid generated previously (Reynard et al. 2011) contains the *GDF5* 5ʹUTR encompassing the rs143383 and rs143384 SNPs ligated into the CpG dinucleotide-free pCpGL luciferase reporter plasmid (Klug and Rehli 2006). Two plasmid haplotypes were generated, one with a C allele at rs143383 and rs143384 (*GDF5*-pCpGL-C) and a second with a T allele at both SNPs (*GDF5*-pCpGL-T). 10 µg of plasmid DNA was in vitro methylated as previously described (Reynard et al. 2011) using the CpG methyltransferase *M.* *SssI* (New England BioLabs, Hitchin, UK). SW1353 chondrosarcoma cells and SW872 liposarcoma cells were seeded as described (Reynard et al. 2011) and co-transfected for 24 h with 500 ng of the appropriate pCpGL vector and 30 ng of the control pRL-TK *Renilla* vector (Promega, Southampton, UK) using ExGen 500 in vitro transfection reagent (Fermentas, York, UK). Luciferase and renilla activity was assayed using the Dual Luciferase Assay System (Promega) and a MicroLUMAT Plus LB96V luminometer (Berthold Technologies). Three independent experiments were performed with six wells transfected per construct.

### EMSAs

Nuclear proteins were extracted from SW872 liposarcoma, SW1353 chondrosarcoma, MG63 osteosarcoma and HACs as previously described (Syddall et al. 2013). Single-stranded DY682-labelled oligonucleotides spanning +26 to +51 relative to the *GDF5* TSS were synthesised by Eurofins MWG Operon and then annealed to generate double-stranded probes (Syddall et al. 2013). Six probe combinations were generated that contained either the C allele or T allele at rs143383 and were unmethylated or methylated at the +37 CpG site and rs143383 CpG site. Binding reactions were performed for 20 min at room temperature using the Odyssey EMSA buffer kit (Licor Biosciences) containing 5 µg nuclear protein, 200 fmol labelled probe, 1× Binding Buffer, 2.5 mM DTT, 0.25 % Tween-20, 1 μg Poly (dI:dC; Invitrogen), and 5 mM MgCl_2_. Samples were separated on a 5 % (w/v) native polyacrylamide gel in 0.5 × TBE for 4 h at 100 V followed by visualisation with the Odyssey infrared imager (Licor Biosciences). For competition assays, unlabelled oligonucleotides identical to the unmethylated probes or oligonucleotides containing the SP1/SP3 transcription factor consensus sequence were added to the binding reaction in excess. All probe and competitor sequences are given in Table 1. For supershift assays, 2 µg of either anti-SP1 (Santa Cruz Biotechnology; sc-59) or anti-SP3 (sc-644) antibody was added to the binding reaction.

### *GDF5*-pCpGL and pEGFP-N1 co-transfections

The SP1, SP3 and SUB1 ORFs were amplified from cDNA and inserted into the *Eco*RI and *Sac*II restriction sites of the pEGFP-N1 plasmid (Clontech) to create SP1-EGFP, SP3-EGFP and SUB1-EGFP plasmids. The DEAF1-EGFP-N1 plasmid was kindly donated by CG Fathman (Yip et al. 2009). The *GDF5* region spanning −93 to +304 relative to the TSS and encompassing the 5ʹUTR was inserted into the *Mlu*I and *Bgl*II sites of the pGL3-enhancer plasmid (Promega) to create rs143383 C and T allele 5ʹUTR-pGL3 enhancer plasmids. Primer sequences are listed in Table 1. 5ʹUTR-pGL3 plasmids were in vitro methylated as above. SW1353 cells were seeded as described (Reynard et al. 2011) and co-transfected with 500 ng of SP1-EGFP or SP3-EGFP plasmid, 500 ng of rs143383 C allele or T allele 5ʹUTR-pGL3 enhancer plasmid and 30 ng of the control pRL-TK *Renilla* vector (Promega, Southampton, UK) using ExGen 500 in vitro transfection reagent. Empty EGFP-N1 and pGL3 enhancer plasmids were used as a control. Five wells were transfected per condition and four independent experiments were performed. After 24 h, luciferase and renilla readings were assayed as above, normalised to the EGFP-N1 and pGL3 control plasmids.

### Statistical analyses

For two-way analyses, a Mann–Whitney *U* test was used. For three-way analyses, a one-way analysis of variants (ANOVA) test was used. Both tests were performed using GraphPad Prism software and the *p* value was Bonferroni corrected to account for multiple testing. For four-way analyses, a two-way ANOVA was performed using SPSS Version 21. An omnibus test was performed testing for methylation, genotype, and their interaction, and if this was statistically significant (*p* value <0.05), pairwise comparisons were performed using Fisher’s Least Significant Difference (LSD) test. Raw *p* values are reported unadjusted for multiplicity.

## Discussion

In this study, we investigated the interaction between genetics, DNA methylation and transcription at the OA susceptibility SNP rs143383. We initially discovered that *GDF5* expression is higher in OA compared to non-OA cartilage, and higher in knee compared to hip cartilage. We then demonstrated that a reduction of DNMT enzyme levels or activity by siRNA knock-down or AZA treatment respectively led to a significant increase in *GDF5* expression in patient chondrocytes, suggesting that DNA methylation regulates *GDF5* expression in the cells that are pivotal to the OA disease process.

DNMT enzymes catalyse the transfer of a methyl group to the cytosine in a CpG dinucleotide to maintain DNA methylation patterns during cell division and to establish new patterns of methylation during development and differentiation (Jones 2012). DNMT1 and DNMT3A predominate in cartilage (Sesselmann et al. 2009), and expression of DNMT1 is reduced in OA cartilage (Hashimoto et al. 2009), although there are no changes in global methylation levels (Sesselmann et al. 2009). Inhibition or loss of DNMT enzymes results in passive demethylation due to failure to methylate the newly synthesised DNA strand to re-establish methylation patterns. DNA methylation inhibits transcription by preventing the binding of methylation-sensitive transcription factors and by the formation of inactive chromatin through the action of methyl-binding proteins and chromatin remodelling complexes. Thus, the increased chondrocyte *GDF5* expression associated with AZA- and siRNA-mediated promoter demethylation presumably results from increased binding of transcription factors that are inhibited by methylation and through prevention of binding by methyl-binding proteins such as MeCP1 and MeCP2. It is also possible that the levels of proteins that regulate *GDF5* expression are altered in these cells, and AZA treatment has previously been reported to decrease levels of SP3, a transcriptional repressor of *GDF5*, in human and mice cells (Lee et al. 2004; Wang et al. 2003).

We next looked at methylation of the *GDF5* CpG island and 5ʹUTR in osteoarthritic cartilage. Changes in promoter methylation have been associated with aberrant expression of several genes in OA cartilage including the transcription factor *SOX9* (Kim et al. 2013), and several matrix degrading enzymes (Roach et al. 2005). We observed increased expression of *GDF5* in OA cartilage and this was accompanied by demethylation of several CpG sites within the *GDF5* CpG island. OA knee cartilage was hypomethylated relative to OA hip and control hip cartilage in both the CpG island and the 5ʹUTR, including the site created by the C allele of rs143383, demonstrating that there are epigenetic differences at the *GDF5* locus between osteoarthritic hip and knee cartilage. To our knowledge, this is the first study to compare methylation patterns between cartilage from different joints and the differences we observed at the *GDF5* locus may reflect global differences in DNA methylation patterns between cartilages from different joints. Such differential methylation by skeletal site could partly explain why there is a limited overlap between the genes aberrantly expressed in OA hip and OA knee cartilage (Xu et al. 2012). The methylation differences between OA and control cartilage in the CpG island and between OA knee and hip cartilage in the 5ʹUTR are only small (3–12 %) but are similar to those observed at the *MMP13* and *SOX9* promoters in OA and control hip cartilage (Bui et al. 2012; Kim et al. 2013). Furthermore, several recent studies have demonstrated that, unlike in cancer, disease-associated methylation differences are small for complex diseases. For example, methylation level differences between monozygotic twins discordant for disease range from 0.3 to 6.6 % in type I diabetes (Rakyan et al. 2011), 4–9 % in schizophrenia and bipolar disorder (Dempster et al. 2011), and from <1–8 % in psoriasis (Gervin et al. 2012).

DNA polymorphisms can influence methylation of nearby CpG sites, leading to differences in methylation levels between alleles, a phenomenon termed allele-specific methylation (ASM; Schalkwyk et al. 2010; Meaburn et al. 2010), with the associated DNA variant referred to as a methylation quantitative trait locus (methQTLs). methQTLs are common in the human genome and may result in allelic differences in gene expression (Zhang et al. 2010; Gibbs et al. 2010; Bell et al. 2011; Tycko 2010). One example is the rs13121031 SNP located within the alternative promoter of the cerebellar ataxia-associated gene *TRPC3*. The C allele of this SNP has decreased methylation of surrounding CpG sites relative to the G allele in several tissues and this correlates with increased expression of the C allele relative to the G allele in brain Martin-Trujillo et al. 2011). It has been proposed that rs143383 may act in a similar manner to rs13121031 to affect methylation of nearby CpG sites, and thus gene expression (Barter and Young 2013). However, we found no evidence that rs143383 genotype affects methylation at any of the CpG sites within the *GDF5* CpG island or 5ʹUTR, although we cannot rule out that rs143383 may influence CpG sites outside of these two regions or that rs143383 may act as an methQTL in other tissues.

In our previous study, we observed that AZA-induced demethylation increased the expression imbalance between the C and T alleles of rs143383, which suggested that DNA methylation can modulate the functional effect of rs143383 on the *GDF5* promoter (Reynard et al. 2011). In the current study, we used in vitro methylation combined with luciferase assays to confirm this observation and to determine if the effect of methylation on rs143383 is direct or indirect. As expected, there was increased promoter activity of the C allele relative to the T allele when the luciferase plasmid was unmethylated. However, hypermethylation of the 5ʹUTR inverted the direction of the imbalance at rs143383 such that the methylated T allele construct had significantly increased promoter activity compared to the methylated C allele construct. Furthermore, this data demonstrated that the effect of methylation on rs143383 is intrinsic to the 5ʹUTR rather than an indirect effect, such as altered expression of *trans*-acting factors.

The methylation of DNA binding sites is known to inhibit the binding of transcription factors (Kim et al. 2013; Bui et al. 2012), with the altered expressions of *MMP13* and *SOX9* in OA hip cartilage resulting from changes in methylation affecting the binding of the transcriptional factor CREB to the *MMP13* and *SOX9* promoters. DNA methylation therefore represents a mechanism that could modulate the allelic expression of rs143383. Using EMSA and overexpression assays, we thus examined the effect of 5ʹUTR methylation on the binding and activity of SP1, SP3, SUB1 and DEAF1, all of which bind to and repress the *GDF5* 5ʹUTR (Syddall et al. 2013).

The SP1/SP3 binding site (GGGGGCGGTC/T) spans both rs143383 and the +37 CpG site located 4 bp upstream, which is significantly hypomethylated in OA knee cartilage. Both proteins bind with higher affinity to the T allele of rs143383 than the C allele in an EMSA assay (Syddall et al. 2013), despite the T allele being more divergent in sequence to the SP1/SP3 consensus binding site of GGGGCGGGG. Methylation of the +37 CpG site, the CpG site created by the C allele at rs143383 or both sites has little or no effect on SP1 or SP3 binding on the background of the C allele at rs143383. However, methylation of the +37 site abrogates the binding of both proteins when the T allele is present at rs143383, such that these proteins bind with less affinity to the methylated T allele than the methylated or unmethylated C allele. These results clearly indicate that the strength of SP1 and SP3 binding to rs143383 is both allele and methylation dependent.

Co-transfection of luciferase constructs with an SP1 overexpression vector confirmed that 5′UTR methylation reduces SP1-mediated transcriptional repression of the T allele of rs143383. However, SP1 repressed the C allele of rs143383 more strongly when methylated; this was unexpected given that methylation of the +37 CpG had no effect on SP1 binding in EMSAs. One explanation for this could be that methylation of additional CpG sites within the 5′UTR outside of the SP1/SP3 site also affect SP1 binding. Surprisingly, overexpression of SP3 repressed both alleles more strongly when methylated than when unmethylated, although the allelic difference in repression was reduced by methylation. Given that SP1 and SP3 compete for binding sites, it is possible that reduced binding of endogenous SP1 to the methylated C and T alleles combined with the increased SP3/SP1 ratio may increase the number of cells in which SP3 can bind over rs143383, even though the affinity of SP3 binding is reduced by methylation. As we have previously reported (Syddall et al. 2013), when the 5ʹUTR is unmethylated, DEAF1 preferentially represses the T allele of rs143383 leading to allelic imbalance in luciferase activity. Methylation abrogates the repressive effects of DEAF1 on the 5′UTR, and affects the T allele more strongly than the C allele causing the abolition of rs143383 imbalance. Whereas SUB1 overexpression has no effect on either allele when unmethylated, methylation resulted in allele-specific affects on promoter activity, with reduced activity of the T allele but increased activity of the C allele. These results indicate that the strength of *trans*-activator binding to and transcriptional repression of rs143383 is both methylation and allele dependent. In light of our findings, we propose the following model. When the +37 site is unmethylated, SP1, SP3 and DEAF1 proteins bind more avidly to the T allele of rs143383, repressing expression of the T allele relative to the C allele, resulting in an imbalance in favour of the C allele similar to that observed in joint tissues. However, when the +37 site is methylated, SP1, SP3 and DEAF-1 binding affinity to the methylated T allele is reduced relative to the methylated C allele. This leads to an increased transcriptional de-repression of the T allele relative to the C, and the allelic imbalance at rs143383 is reduced or even reversed.

Numerous studies have shown that the effect of DNA methylation on binding of SP1 and SP3 to the DNA is dependent on the binding site sequence, interacting proteins and cell type. Methylation has no effect on SP1 binding to the *CLDN4* promoter (Honda et al. 2006) or SP1/SP3 consensus binding site (Höller et al. 1988), whereas methylation within the consensus sequence inhibits SP1 binding to the *Cadm1* gene *(*Reamon-Buettner and Borlak 2008), and methylation adjacent to, but not within the SP1 site, reduces SP1 binding at the *p21* ^*Cip1*^ promoter (Zhu et al. 2003). Methylation also has variable effects on SP3 binding affinity, with methylation inhibiting binding of SP3 to the chondrocyte-specific *ChM*-*1* promoter (Aoyama et al. 2004), leading to gene repression. In contrast, methylation increases binding of SP3 and the methyl-binding protein MBD2 to the mouse δ-Opioid receptor (*mDOR*) promoter (Wang et al. 2003), and SP3 can recruit chromatin remodelling proteins leading to gene repression (Won et al. 2002). As well as altering binding affinity, methylation can also alter the function of SP3, with SP3 increasing the SP1-mediated activity of the unmethylated *mDOR* promoter but inhibiting SP1-mediated promoter activity of the methylated *mDOR* promoter (Wang et al. 2003). A similar mechanism may occur at rs143383, with methylation increasing the repressive activity of SP3 despite reducing SP3 binding affinity, and this could explain why SP3 represses the methylated 5′UTR luciferase plasmid more strongly than the unmethylated plasmid.

In our previous study, we suggested that differences in *GDF5* 5ʹUTR DNA methylation could explain the intra- and inter-individual differences in allelic imbalance of rs143383 observed in joint tissues from individuals with OA. In the present study, we have uncovered the molecular mechanism by which DNA methylation modulates the allelic expression of rs143383 and discovered that OA knee cartilage is hypomethylated relative to both normal and OA hip cartilage. These differences in 5ʹUTR methylation, especially at the +37 CpG site, could explain why rs143383 is an OA susceptibility allele particularly for the knee compared to the hip in Europeans. In addition, differences in methylation levels between different joint tissues could underlie the different odds ratios (ORs) for the different pathologies associated with rs143383. The OR for rs143383 in knee OA is 1.17 but the SNP has larger effect sizes on congenital hip dysplasia (OR 1.37; Rouault et al. 2010) and lumbar disc degeneration (OR 1.72; Williams et al. 2011)). Furthermore, ethnic differences in *GDF5* methylation as a result of different environmental and genetic factors could account for the disparity between the ORs of rs143383 in European and Asians; rs144383 has an OR of 1.53 in knee OA in a Thai population (Tawonsawatruk et al. 2011) and an OR of 1.79 in a Japanese cohort (Chapman et al. 2008). One area of future research to confirm these hypotheses will therefore be to examine *GDF5* 5ʹUTR methylation and AEI of rs143383 in joint tissues from the different pathologies, and in cartilage from OA patients of different ethnic backgrounds.

To conclude, we have found that the OA susceptibility gene *GDF5* is regulated by DNA methylation in cartilage from patients and that aberrant methylation correlates with increased *GDF5* expression. Methylation of the 5ʹUTR modulates the functional effects of the OA polymorphism rs143383 on *GDF5* gene expression through interfering with the binding of transcriptional repressor proteins including SP1 and SP3 in an allele-specific manner. Furthermore, the +37 CpG site within the SP1 and SP3 binding site is hypomethylated in knee cartilage relative to hip cartilage and we hypothesise that differential methylation of this site may be responsible for the joint specific effect of the rs143383 SNP on OA susceptibility.

## Electronic supplementary material

Below is the link to the electronic supplementary material.

Supplementary material 1 Supplemental data comprise two figures. Supplemental Fig. 1. **(A)** *GDF5* expression in NOF and OA hip cartilage assayed by microarray analysis using the data of Xu et al. (2012). ***p < 0.001, Mann–Whitney *U* test. **(B) to (D)** *GDF5* expression assayed in cartilage by qRT-PCR and stratified by **(B)** age, **(C)** gender, and **(D)** genotype at the OA-associated rs143383 (TIFF 6325 kb)

Supplementary material 2 (A) Correlation between *GDF5* gene expression and methylation of the CpG island (CGI) in cartilage. **(B)** and **(C)** Total CpG island methylation in cartilage from (B) females and (C) males. **(D)** and **(E)** Total 5ʹUTR methylation in female and male cartilage, respectively. **(F)** and **(G)** effect of rs143383 genotype on CpG island and 5ʹUTR methylation, respectively, in cartilage from NOF, OA hip and OA knee patients. Methylation was assessed by pyrosequencing, the bar in A-E represents the mean and the error bars in F and G denote standard error. * p < 0.05, ***p < 0.001, one-way ANOVA with Bonferroni correction (TIFF 12922 kb)

Supplementary material 3 EMSA of SW872 nuclear protein lysates with unmethylated rs143383 T and C probes. Increasing concentrations of non-labelled T and C allele competitors were added to the binding reaction. Higher concentrations of competitor are required to reduce formation of the T allele-protein complexes than the C allele-protein complexes (TIFF 2420 kb)
