# Supplementary figures and images for: CpG methylation regulates allelic expression of GDF5 by modulating binding of SP1 and SP3 repressor proteins to the osteoarthritis susceptibility SNP rs143383
Source: Hum Genet. 2014 May 27;133(8):1059–73. doi: 10.1007/s00439-014-1447-z (PMC4099533; doi:10.1007/s00439-014-1447-z)

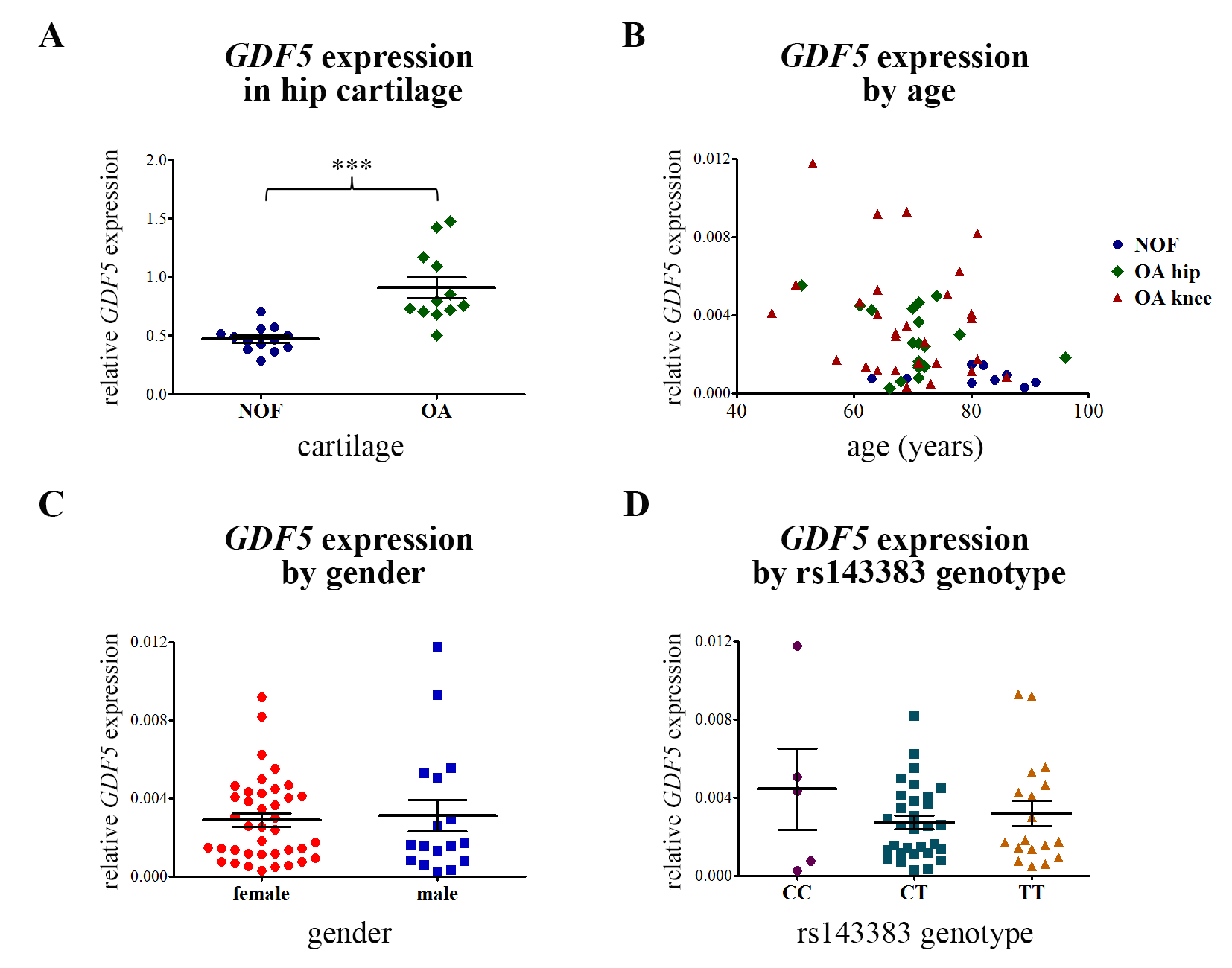

Supplement: Supplementary file 1 — Supplemental data comprise two figures. Supplemental Fig. 1. (A) GDF5 expression in NOF and OA hip cartilage assayed by microarray analysis using the data of Xu et al. (2012). ***p < 0.001, Mann–Whitney U test. (B) to (D) GDF5 expression assayed in cartilage by qRT-PCR and stratified by (B) age, (C) gender, and (D) genotype at the OA-associated rs143383 (TIFF 6325 kb) [file 439_2014_1447_MOESM1_ESM.tif]

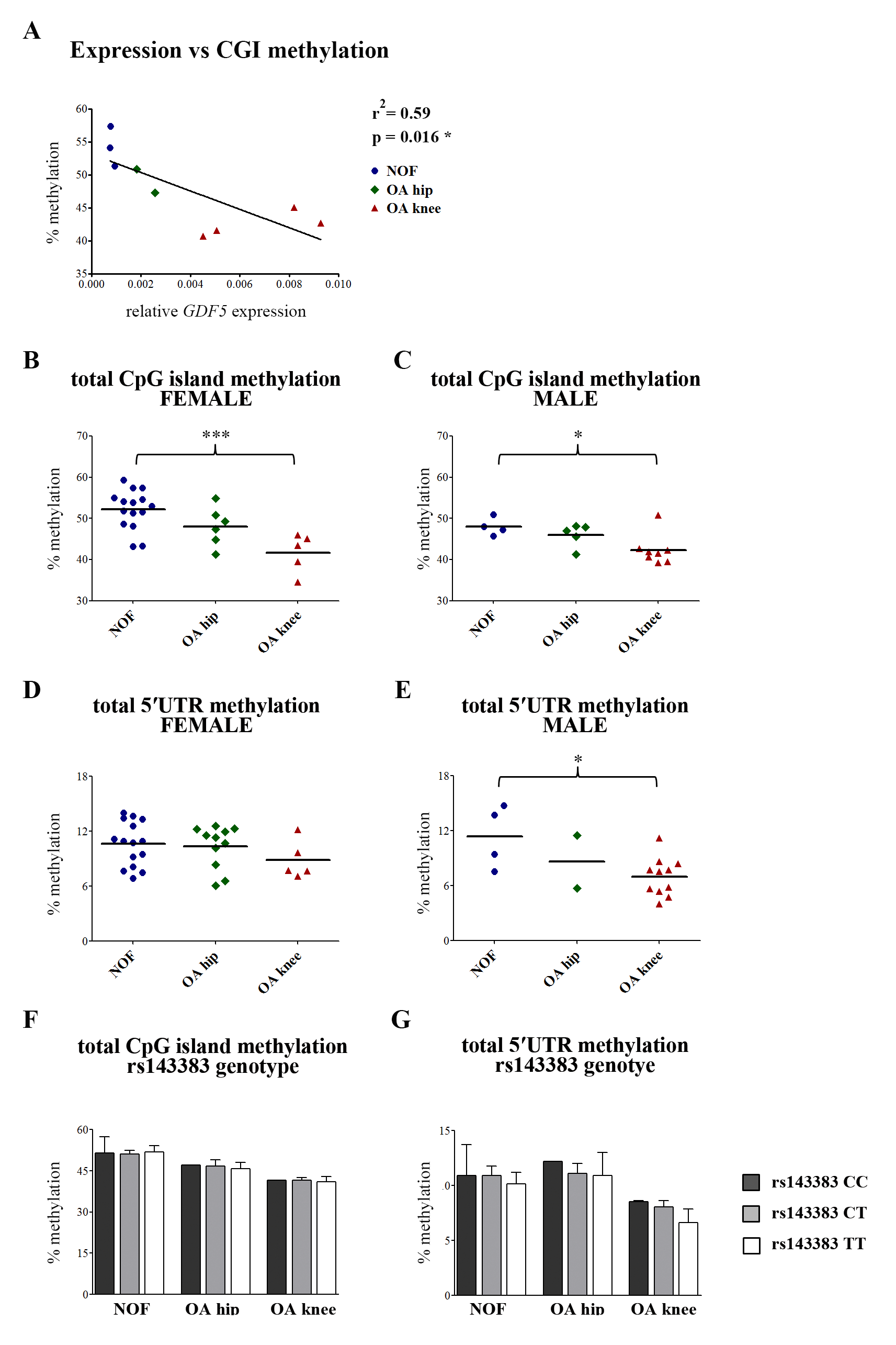

Supplement: Supplementary file 2 — (A) Correlation between GDF5 gene expression and methylation of the CpG island (CGI) in cartilage. (B) and (C) Total CpG island methylation in cartilage from (B) females and (C) males. (D) and (E) Total 5ʹUTR methylation in female and male cartilage, respectively. (F) and (G) effect of rs143383 genotype on CpG island and 5ʹUTR methylation, respectively, in cartilage from NOF, OA hip and OA knee patients. Methylation was assessed by pyrosequencing, the bar in A-E represents the mean and the error bars in F and G denote standard error. * p < 0.05, ***p < 0.001, one-way ANOVA with Bonferroni correction (TIFF 12922 kb) [file 439_2014_1447_MOESM2_ESM.tif]

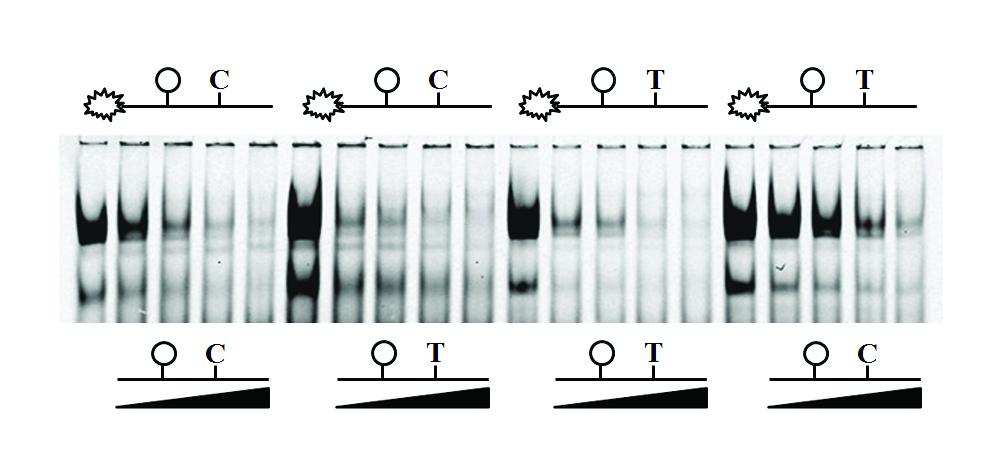

Supplement: Supplementary file 3 — EMSA of SW872 nuclear protein lysates with unmethylated rs143383 T and C probes. Increasing concentrations of non-labelled T and C allele competitors were added to the binding reaction. Higher concentrations of competitor are required to reduce formation of the T allele-protein complexes than the C allele-protein complexes (TIFF 2420 kb) [file 439_2014_1447_MOESM3_ESM.tif]
